# A Plant Germline-Specific Integrator of Sperm Specification and Cell Cycle Progression

**DOI:** 10.1371/journal.pgen.1000430

**Published:** 2009-03-20

**Authors:** Lynette Brownfield, Said Hafidh, Michael Borg, Anna Sidorova, Toshiyuki Mori, David Twell

**Affiliations:** 1 Department of Biology, University of Leicester, Leicester, United Kingdom; 2 Miyagishima Initiative Research Unit, Advance Science Institute, RIKEN, Wako, Saitama, Japan; The University of North Carolina at Chapel Hill, United States of America

## Abstract

The unique double fertilisation mechanism in flowering plants depends upon a pair of functional sperm cells. During male gametogenesis, each haploid microspore undergoes an asymmetric division to produce a large, non-germline vegetative cell and a single germ cell that divides once to produce the sperm cell pair. Despite the importance of sperm cells in plant reproduction, relatively little is known about the molecular mechanisms controlling germ cell proliferation and specification. Here, we investigate the role of the *Arabidopsis* male germline-specific Myb protein DUO POLLEN1, DUO1, as a positive regulator of male germline development. We show that DUO1 is required for correct male germ cell differentiation including the expression of key genes required for fertilisation. DUO1 is also necessary for male germ cell division, and we show that DUO1 is required for the germline expression of the G2/M regulator AtCycB1;1 and that AtCycB1:1 can partially rescue defective germ cell division in *duo1*. We further show that the male germline-restricted expression of DUO1 depends upon positive promoter elements and not upon a proposed repressor binding site. Thus, DUO1 is a key regulator in the production of functional sperm cells in flowering plants that has a novel integrative role linking gametic cell specification and cell cycle progression.

## Author Summary

Flowering plants, unlike animals, require not one, but two sperm cells for successful fertilisation—one sperm cell to join with the egg cell to produce the embryo and the other to join with the central cell to produce the nutrient-rich endosperm tissue inside the seed. A mystery in this “double fertilization” process was how each single pollen grain could produce the pair of sperm cells needed for fertility and seed production. Here, we report the discovery of a dual role for *DUO1*, a regulatory gene required for plant sperm cell production. We show that the *DUO1* gene is required to promote the division of sperm precursor cells, while at the same time promoting their differentiation into functional sperm cells. DUO1 is required for the expression of a key cell cycle regulator and for the expression of genes that are critical for gamete differentiation and fertilisation. This work provides the first molecular insight into the mechanisms through which cell cycle progression and gamete differentiation are coordinated in flowering plants. This knowledge will be helpful in understanding the mechanisms and evolution of gamete development in flowering plants and may be useful in the control of gene flow and crossing behaviour.

## Introduction

The gametes of flowering plants are formed by discrete haploid gametophyte structures consisting of only a few cells that develop within the diploid reproductive floral organs. During spermatogenesis, each single haploid microspore divides asymmetrically to produce a larger vegetative cell that eventually gives rise to the pollen tube and a smaller germ, or generative, cell (Figure S1; reviewed in [1],[2]). In contrast to germline cells in metozoans [3], angiosperm male germ cells do not undergo regenerative stem cell divisions, but divide once to form a pair of sperm cells. These sperm cells are delivered to the embryo sac via the pollen tube, where they fuse with egg and central cells to produce embryo and endosperm respectively. This process of double fertilization depends upon two functional sperm cells and is considered one of the major advances in the evolutionary success of flowering plants. Despite this importance, the molecular mechanisms underlying many component processes, including the production of both male and female gametes, remain largely unknown.

Recent transcriptomic analysis of isolated *Arabidopsis* sperm cells shows that sperm cells express a distinct and diverse set of genes [4] and there is evidence for extensive male germ cell gene expression in maize and lily [5],[6]. Several male germline-specific genes have been characterized in *Arabidopsis* including *AtMGH3*, encoding a histone H3.3 variant [7],[8], *AtGEX2*, encoding a putative membrane associated protein [9], and *AtGCS1* (*HAP2*), encoding a sperm cell surface protein required for fertilisation [10],[11]. Homologues of *AtGCS1* are found in many genera [5],[12],[13] that include the green alga *Chlamydomonas* and the rat malarial parasite *Plasmodium berghei*, where they are required for gamete interactions and membrane fusion [13]. Although gene expression in angiosperm sperm cells is extensive and essential for gamete functions little is known about its regulation. A transcriptional derepression mechanism, in which expression of male germline expressed genes is repressed in all non-germline cells by a protein called Germline Restrictive Silencing Factor (GRSF), has recently been proposed [14]. A binding site for the GRSF protein was identified in the promoter region of the Lily male germline gene *LGC1*, and mutations in this sequence led to the ectopic activation of the *LGC1* promoter in non-germline cells in lily and *Arabidopsis*. Although similar binding sites have been found in the promoter regions of several germline genes in *Arabidopsis*, including the germline-specific transcription factor gene *DUO1* [14], a functional role for these sites or of GRSF activity in regulating gene expression in *Arabidopsis* pollen has not been shown.

Germ cell division resulting in the sperm cell pair in each pollen grain, is essential for double fertilization and recent data supports the capacity of both sperm cells to fertilize the egg cell in *Arabidopsis* [15]. Several mutants have been described in which germ cell division is disrupted [16]–[18]. Mutations in the conserved cell cycle regulator CDKA1 [16],[17] and in the F-BOX protein FBL17 [18] prevent germ cell division and result in mature pollen with a single germ cell. Defects in Chromosome Assembly Factor 1 (CAF1) can also disrupt germ cell division [19]. Interestingly, the single germ cells in these mutants are capable of fertilization, with *cdka1* and *fbl17* mutant germ cells fertilizing the egg cell to produce an embryo that aborts early in development due to the lack of endosperm production. These mutations clearly demonstrate that germ cell division and specification can be uncoupled, but do not identify how these processes may be coordinated to produce twin sperm cells competent for double fertilization.

DUO POLLEN1 (DUO1) is a unique male germ cell-specific R2R3 Myb protein that is also required for germ cell division in *Arabidopsis* [20]. Unlike *cdka1* and *fbl17* single germ cells, *duo1* germ cells do not lead to successful fertilization, suggesting that in addition to germ cell cycle defects, key features of gamete differentiation and function are impaired in *duo1*. Here we further characterize DUO1 as an essential, positive regulator of sperm cell production in plants. We use various molecular markers and ectopic expression assays to show that DUO1 is both necessary and sufficient for the expression of male germline genes. We show that DUO1 is required for the expression of the *Arabidopsis* G2/M regulator CyclinB1;1 (AtCycB1;1) in the male germline and that AtCycB1:1 can partially rescue defective germ cell division in *duo1*. Our findings reveal a novel integrative role for the germline-specific DUO1 protein, in cell specification and cell cycle progression necessary for twin sperm cell production. Furthermore, we show that restriction of *DUO1* expression to the male germline is not dependent on a putative GRSF binding site but involves positive elements in the promoter.

## Results/Discussion

### DUO1 Is a Key Regulator of Sperm Cell Specification

**Figure 1:**
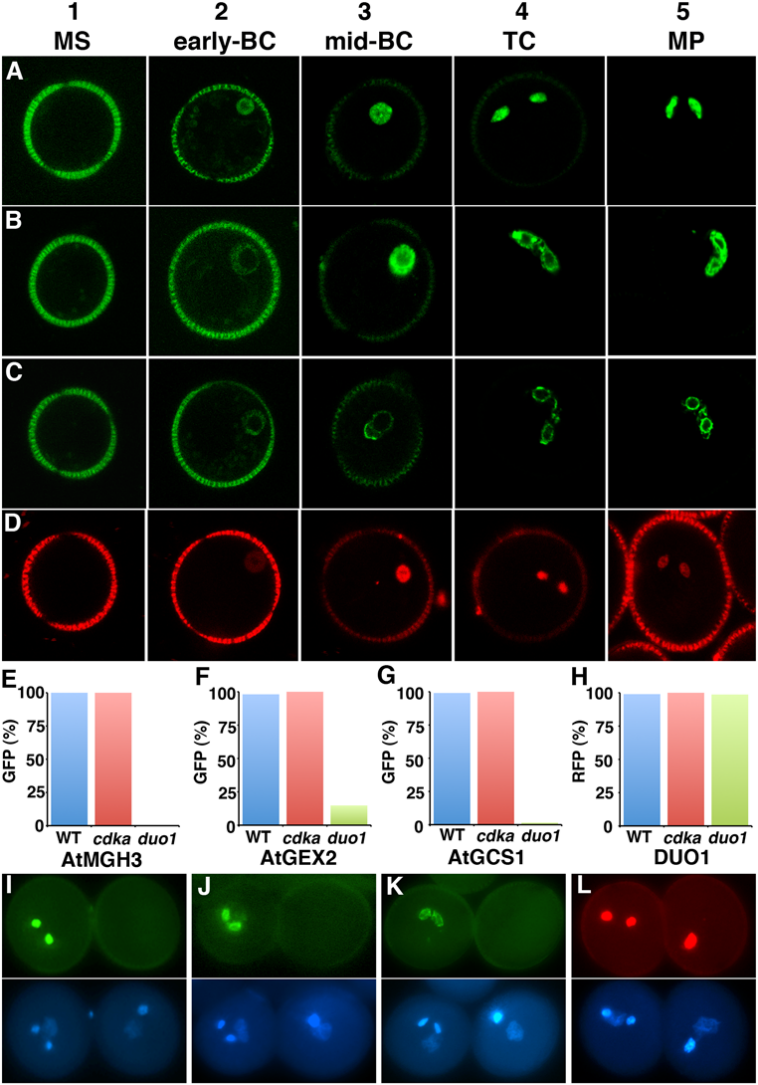
Expression of male germline-specific genes in wild type and duo1 pollen. Expression of AtMGH3-H2B::GFP (A), AtGEX2-GFP (B), AtGCS1-AtGCS1::GFP (C) and DUO1-DUO1::mRFP (D) during wild type pollen development, observed with CLSM. Panels are numbered 1 (left) to 5 (right). For all markers, fluorescence is not detected in microspores (MS; Panel 1), a weak signal is detected in the germ cell during or soon after engulfment (early-BC; Panel 2), fluorescence increases in mid-bicellular pollen (mid-BC; Panel 3) and remains in tricellular (TC; Panel 4) and mature pollen (MP; Panel 5). (E–L) Expression of germline expressed genes in heterozygous *duo1* plants. The percentage pollen showing GFP or RFP in sperm cells of wild type (WT) pollen or the single germ cell in *cdka;1* and *duo1* mutant pollen in plants homozygous for AtMGH3-H2B::GFP (AtMGH3, E), AtGEX2-GFP (AtGEX2, F), AtGCS1-AtGCS1::GFP (AtGCS1, G) and DUO1-H2B::mRFP (DUO1, H). Individual examples viewed by fluorescence microscopy in I to L. AtMGH3-H2B::GFP (I), AtGEX2-GFP (J) and AtGCS1-AtGCS1::GFP (K) are not expressed, or have reduced expression in *duo1* pollen while DUO1-H2B::RFP (L) is expressed. Each image has a wild type pollen grain to the left and a *duo1* mutant grain to the right (see lower DAPI images).

To investigate the potential role of DUO1 in regulating sperm specification we examined the expression of three male germline markers, *AtMGH3*, *AtGEX2* and *AtGCS1*, in mutant *duo1* pollen. We exploited marker lines with promoter regions of these germline genes linked to GFP. First we characterised the expression of these markers in a coordinated manner using confocal laser scanning microscopy (CLSM) throughout development of wild-type pollen (Figure 1A–C), and compared their profiles with the expression of a DUO1∶mRFP fusion protein under control of the *DUO1* promoter (DUO1-DUO1::mRFP; Figure 1D). The expression of all three germ cell markers is undetectable in free microspores when DUO1 is not expressed (Figure 1, Panel 1). Fluorescence is first detected in the germ cell during or soon after engulfment by the vegetative cell, appearing at a similar time to the expression of DUO1 (Figure 1, Panel 2). As the pollen matures the level of GFP accumulates in germ cells before mitosis and remains high in mature sperm cells (Figure 1A–C, Panels 3–5). The accumulation of GFP in progressive stages is illustrated by the reduced autofluorescence signal arising from the pollen wall, reflecting the reduced exposure needed to capture a relatively unsaturated germ cell GFP signal. DUO1 expression persists during pollen development, although its abundance does not obviously increase in tricellular and mature pollen (Figure 1D). Our analysis shows that in common with *AtMGH3* and *AtGEX2*, the expression of *AtGCS1*, previously thought to be sperm cell-specific in *Arabidopsis* [11], is detected in germ cells soon after asymmetric division (Figure 1C). The expression of all male germ cell markers shortly after the asymmetric division shows that sperm cell specification begins early after inception of the germline prior to passage of germ cells through mitosis.

The three male germline markers were introduced into heterozygous *duo1* plants that produce 50% wild type pollen and 50% mutant pollen, and GFP expression was scored. Virtually all the wild type pollen showed GFP fluorescence in twin sperm cells while there was no fluorescence, or rarely a weak GFP signal, in the single germ cell in *duo1* pollen (Figure 1E–G, I–K; Table S1). When these markers were introduced into the *cdka;1* mutant in which the arrested germ cell is able to fertilize the egg cell, fluorescence was observed in the single germ cells in mutant pollen (Figure 1E–G, Table S1). This result confirms that germ cell division and cell fate specification are uncoupled in *cdka;1* mutant pollen, similar to the observed expression of germ cell markers in arrested but functional germ cells in *CAF1* mutants [19]. The absence of GFP in mutant *duo1* germ cells demonstrates that DUO1 is necessary for the expression of several germline-expressed genes, and explains why *duo1* pollen is infertile (it lacks proteins including AtGCS1 that are essential for fertilization). In contrast, when the *DUO1* promoter was used to express a nuclear-targeted histone H2B::mRFP marker protein, fluorescence was detected in mutant *duo1* germ cells, similar to its expression in wild type sperm cells and in *cdka;1* germ cells (Figure 1H,L; Table S1), indicating that *DUO1* promoter activation does not depend upon DUO1 itself.

**Figure 2:**
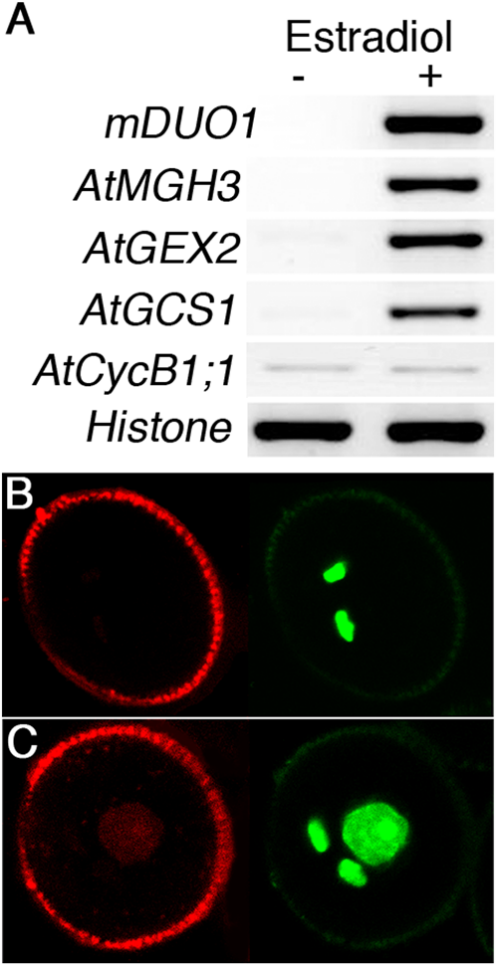
Ectopic expression of DUO1 results in expression of male germline specific genes. (A) RT-PCR analysis of *mDUO1*, *AtMGH3*, *AtGEX2*, *AtGCS1* and *AtCycB1;1* expression in whole seedlings transformed with the *mDUO1* cDNA (see methods) under the control of an estradiol inducible promoter grown on media without estradiol (−) or with estradiol (+). Histone H3 was used as a control. (B, C) Mature pollen grains showing AtMGH3-H2B::GFP expression specifically in sperm cells in the absence of LAT52-DUO1::mRFP (B), or in both the vegetative cell nucleus and sperm cells in the presence of LAT52-DUO1::mRFP (C). Left and right panels correspond to RFP and GFP signals viewed by CLSM.

To independently confirm the regulation of germline genes by DUO1 we ectopically expressed DUO1 in seedlings, and in pollen vegetative cells, where *AtMGH3*, *AtGEX2* and *AtGCS1* are not normally expressed. As *DUO1* contains a recognition site for microRNA159 we used a resistant *DUO1* cDNA (*mDUO1*) with an altered nucleotide sequence at the miR159 binding site, but encoding the native amino acid sequence [21]. Transgenic seedlings in which the m*DUO1* cDNA was placed under the control of an estradiol inducible promoter [22] showed *mDUO1* induction when exposed to estradiol (Figure 2A). Expression of the male germline genes, *AtMGH3*, *AtGEX2* and *AtGCS1*, was also induced, with high levels of transcripts present only in plants exposed to estradiol and containing *mDUO1* (Figure 2A). Similarly, when a DUO1::mRFP fusion was ectopically expressed in pollen vegetative cells using the *LAT52* promoter [23], we observed ectopic expression of the *AtMGH3* marker in vegetative cell nuclei (Figure 2B,C; Table S2). Thus ectopic expression of DUO1 is sufficient for activation of germ cell-specific gene expression in a range of non-germline cells.

### DUO1 Is Required for AtCycB1;1 Expression in the Male Germline

**Figure 3:**
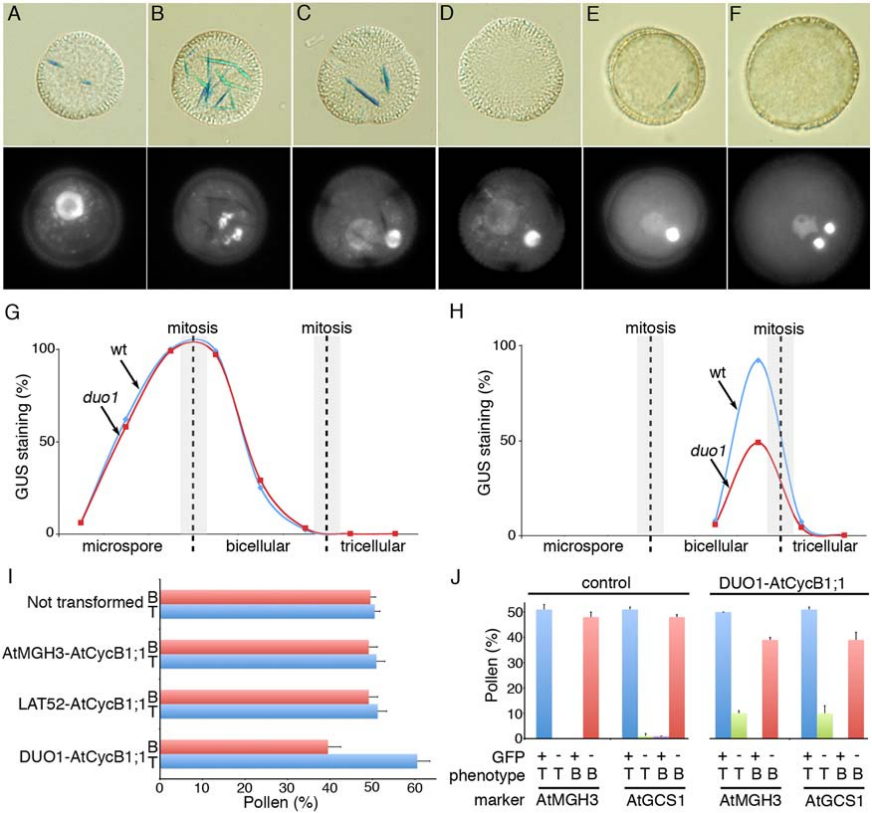
*AtCycB1;1* expression in developing pollen. (A–F), pCDG-dependent GUS staining (upper panel) and DAPI staining (lower panel) in isolated spores: (A, B), unicellular microspores, (C, D, E), early, mid-and late bicellular pollen and (F), tricellular pollen. (G, H) The frequency of pCDG-dependent GUS staining in microspores and vegetative cells close to mitosis is similar in *duo1* heterozygotes and wild type plants (G), whereas GUS staining in germ cells, is reduced by approximately half in *duo1* heterozygotes, where 50% of the pollen is WT and the other 50% mutant (H). The stage of pollen development is indicated below each graph and the approximate time of mitosis is indicated by grey squares with a dashed line. (I) DUO1-AtCycB1;1 is able to partially complement the bicellular phenotype of *duo1* pollen. The amount of tricellular pollen (T) increases and the amount of bicellular pollen (B) decreases when heterozygous *duo1* plants are transformed with DUO1-AtCycB1;1 (n = 31 T1 lines) compared with plants either not transformed (n = 3 individuals) or transformed with control constructs AtMGH3-AtCycB1;1::GFP (n = 17 T1 lines) or LAT52-AtCycB1;1 (n = 17 T1 lines). Bars represent the average percentage of pollen with error bars showing standard deviation. (J) Germline markers are not activated in the complemented tricellular pollen. In non-complemented plants ∼50% of the pollen is tricellular (T) with marker expression and ∼50% is bicellular (B) without marker expression. When the bicellular phenotype is partially complemented by DUO1-AtCycB1;1, ∼10% of pollen is tricellular without marker expression, while there is a decrease in the amount of bicellular pollen. Bars represent the average percentage of pollen from 3–6 individual plants with the error bars showing standard deviation.

The phenotype of *duo1* shows that in addition to the activation of male germline genes, DUO1 is required for germ cell division. Mutant *duo1* germ cells complete DNA synthesis (S) phase but fail to enter mitosis (M) [20],[24], suggesting that DUO1 may regulate the expression of essential G2/M factors. As the *Arabidopsis* CDK regulatory subunit AtCycB1;1 shows enhanced expression at G2/M [25],[26] and is expressed in developing pollen (Figure S2), we investigated *AtCycB1;1* as a potential downstream target of DUO1. To monitor the expression of AtCycB1;1 we used the pCDG marker which contains the *AtCycB1;1* promoter region and mitotic destruction box fused to the β-glucuronidase (GUS) reporter [25]. First we analysed the marker in wild type pollen (Figure 3A–F). Individual pollen grains at different stages of development (as determined by DAPI staining) were analysed for GUS activity, which results in the formation of indigo microcrystals. Microspores and bicellular pollen shortly after mitosis contain numerous indigo crystals, with the number peaking close to mitosis (Figure 3A–C), indicating that expression of AtCycB1;1 is linked to asymmetric division. Expression is then abolished in bicellular pollen (Figure 3D). Close to germ cell mitosis, single indigo crystals are present specifically in germ cells (located by DAPI staining; Figure 3E) indicating expression of AtCycB1;1 in the germ cell before division. The protein is degraded after mitosis and is absent in tricellular pollen (Figure 3F).

We then counted the number of pollen grains with GUS staining at different stages of development in wild type and heterozygous *duo1* plants. In both wild type and heterozygous *duo1* plants, polarized microspores and vegetative cells shortly after asymmetric division showed almost 100% staining, indicating expression of AtCycB1:1 (Figure 3G). Thereafter vegetative cell staining declined and was absent from late-bicellular stage pollen (Figure 3G). Germ cell staining was subsequently observed in ∼100% of pollen from wild type plants close to mitosis, but was reduced by approximately half in heterozygous *duo1* plants at this stage (Figure 3H). As half of the pollen population is mutant in heterozygous *duo1* plants, and wild type pollen show GUS staining, this reduction in staining is consistent with a lack of AtCycB1;1 expression in mutant *duo1* pollen. This indicates that DUO1 is required for the expression of AtCycB1;1 in male germ cells.

We then analysed the expression of *AtCycB1;1* transcripts in seedlings after steroid induction of *mDUO1*. In contrast to the germline markers, *AtCycB1;1* was expressed at a low level in seedlings not exposed to estradiol and the presence of DUO1 did not affect the level of *AtCycB1;1* transcripts (Figure 2A). Thus, although DUO1 is required for germline expression of AtCycB1;1 the presence of *DUO1* is not sufficient to induce *AtCycB1;1* mRNA in seedlings. Transcription of the *AtCycB1;1* gene is known to be regulated by a number of factors, including activators such as three repeat [27] or other Myb proteins [28] and TCP20 [29] and repressors such as TOUSLED [30]. Thus, DUO1 may be unable to overcome these controls in seedlings, and may affect *AtCycB1;1* transcription in the male germline through an indirect mechanism or through effects on AtCycB1;1 protein stability.

To investigate the role of AtCycB1;1 in the failure of *duo1* male germ cells to enter mitosis we determined whether AtCycB1;1 is sufficient to rescue the germ cell mitosis defect in *duo1* pollen. We used the *DUO1* promoter to drive *AtCycB1;1* expression in the male germline. The proportion of bicellular or tricellular pollen grains from heterozygous *duo1* plants either not transformed or transformed with either of two control constructs (MGH3-AtCycB1;1::GFP, which is not expressed in mutant pollen, and LAT52-AtCycB1;1, which is expressed only in the vegetative cell) did not vary significantly from 50% (Chi2 p<0.05) (Figure 3I, Table S3). In contrast, in heterozygous *duo1* plants transformed with DUO1-AtCycB1:1 the majority of lines (31/49) showed a significantly reduced frequency of bicellular pollen and a corresponding increase in tricellular pollen (Figure 3I, Table S3). This suggests that restoring AtCycB1;1 in *duo1* mutant germ cells is sufficient to promote mitosis in a proportion of the population. Complementation was however incompletely penetrant, which may result from the use of the *DUO1* promoter that may not produce native amounts of AtCycB1;1. It is also possible that other factors with a role in G2/M transition, such as other AtCycB family members that are also expressed during pollen development [31], may also be absent in *duo1* pollen.

To determine if the presence of DUO1-AtCycB1;1 in *duo1* pollen restored only the ability to proceed through mitosis or germline specification as well, we analysed expression of the *AtMGH3* and *AtGCS1* markers in *duo1* plants showing partial complementation (Figure 3J, Table S4). In contrast to plants without DUO1-AtCycB1;1 where almost all tricellular pollen expresses GFP, plants displaying partial complementation produce ∼10% of pollen that is tricellular but does not express the markers. As there is also a ∼10% decrease in bicellular pollen, this new class of tricellular pollen is most likely *duo1* pollen in which the division defect has been complemented by the DUO1-AtCycB1;1 construct, but in which the markers have not been activated. Consistent with this, DUO1-AtCycB1;1 complemented *duo1* pollen showed no male transmission (Table S5). Thus, complementation of the bicellular phenotype by AtCycB1;1 only affects cell division and does not restore expression of germline gene expression and sperm cell function.

### DUO1 Expression Is Restricted to the Male Germline Independent of a Putative GRSF Binding Site

Closer examination of mature pollen grains ectopically expressing DUO1 in the vegetative cell revealed a distinctive morphology with reduced cytoplasmic density, larger vacuoles and numerous large cytoplasmic inclusions (Figure S3). This phenotype was only found in pollen containing vegetative nucleus GFP (Table S6) and analysis of pollen viability revealed up to 50% non-viable pollen with the aberrant pollen not being viable (Figure S3E, F, Table S7). Similar phenotypes are not seen in pollen of plants transformed with LAT52-H2B::GFP where the transgene is transmitted normally (data not shown). Furthermore, *Arabidopsis* plants constitutively expressing DUO1 (driven by the 35S promoter) show severe seedling patterning defects, twisted and curled leaves and floral defects [21]. These phenotypes demonstrate the importance of restricting high level expression of DUO1 to male germ cells.

**Figure 4:**
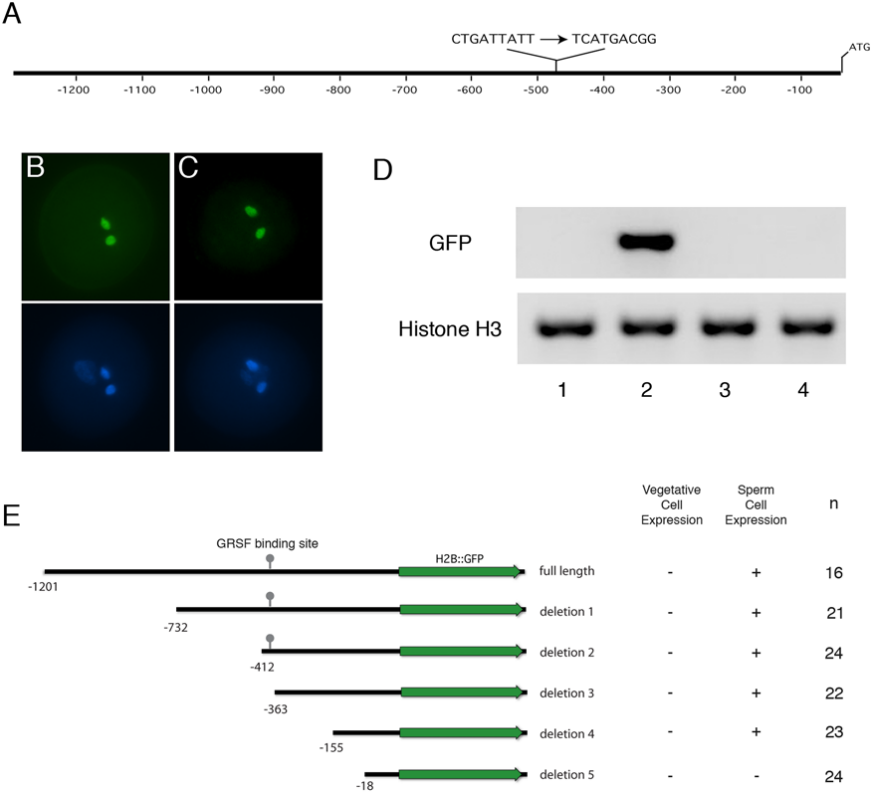
Male germline specificity of DUO1 does not depend on putative GRSF binding sites. (A) Schematic of the *DUO1* promoter region illustrating the mutagenized putative GRSF binding site. (B,C) Expression of H2B::GFP in pollen driven by the native (B) or mutagenized *DUO1* (C) promoters. Top panels show GFP signal, lower panels show DAPI staining. (D) RT-PCR analysis of native and mutagenized DUO1 promoter activity in seedlings. PCR was conducted on cDNA from wild type plants (1), control plants transformed with a constituitive HistoneH3 promoter-H2B::GFP fusion (2), and plants transformed with the native (3), or mutagenized (4), *DUO1* promoters driving H2B::GFP expression. The primers used were specific for GFP (upper panel) or native Histone H3 transcripts (lower panel). The native or mutagenized *DUO1* promoters showed no sporophytic expression of GFP transcripts. (E) Schematic representation of the of the *DUO1* promoter 5′ deletion series used to drive expression of H2B::GFP. The first four deletions, including deletion 3 in which the putative GRSF binding site is removed, showed a similar expression pattern to that of the full-length DUO1 promoter, with GFP signal only observed in sperm cell nuclei. The same expression pattern was observed in all independent lines examined (n). GFP expression was not observed in any transformants harbouring the shortest promoter fragment (deletion 5).

Such restriction may partially rely upon degradation of *DUO1* mRNA by microRNA159 [21] in certain cell types but promoter elements are also likely to be important. As such, restriction of *DUO1* expression to the male germline has been proposed to rely on the repressor protein GRSF due to a putative GRSF binding site in the *DUO1* promoter [14]. Mutagenesis of similar sequences in the *LGC1* promoter led to ectopic activation of the *LGC1* promoter in non-germ line cells in tobacco and *Arabidopsis* [14]. However, when we specifically mutated the putative GRSF binding site in the *DUO1* promoter this did not affect the germline-specific expression of *DUO1* (Figure 4A–D). Moreover, sequences in the 150 bp proximal *DUO1* promoter, excluding putative GRSF binding sites, were sufficient for germline-specific expression (Figure 4E). Although factors that bind to the lily LGC1 silencer appear to be present in non-germline cells in *Arabidopsis* [14], the germline-restricted activation of *DUO1* does not appear to involve GRSF mediated repression. Since the *DUO1* promoter appears to be active only after asymmetric division in the newly formed germ cell and that activation does not depend upon DUO1 itself (see Figure 1), activation of the *DUO1* promoter may depend on proximal region-binding transcription factors that are inherited and/or segregated during asymmetric division of the microspore.

### Conclusions

**Figure 5:**
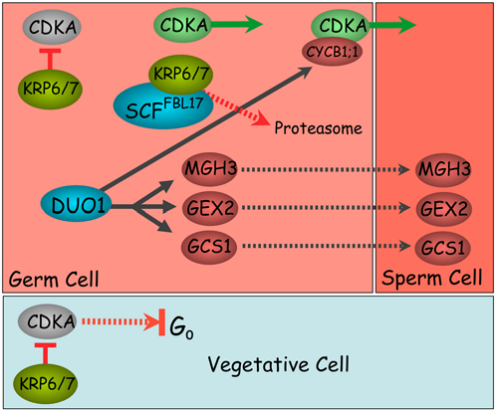
Regulatory events in plant male germ cell production and specification. Model integrating the role of DUO1 and SCF^FBL17^ [18] in plant germ cell production and specification. The germline-specific DUO1 protein (blue) activates the expression of several germline specific proteins (red). In parallel, the CDKA inhibitors KRP6 and KRP7 (green) are expressed in the vegetative cell and germ cell after asymmetric division, where they inhibit CDKA activity and S phase progression. The F-box protein FBL17 is then transiently expressed in the germline and forms an SCF^FBL17^ complex (blue) that targets KRP6/7 for proteasome dependent proteolysis, licensing S-phase progression (green arrow). Further germ cell cycle progression is controlled by the DUO1-dependent G2/M phase expression of the CDKA regulatory subunit AtCYCB1;1 (red). Thus, while SCF^FBL17^ and DUO1 promote male germ cell proliferation at successive stages of the cell cycle, DUO1 integrates germ cell specification and division to ensure the production of functional twin sperm cells that are essential for double fertilization. Arrows indicate a requirement for the protein rather than direct binding.

We have shown that DUO1 is both necessary and sufficient for the expression of several male germline genes including *AtGCS1* that is required for gamete fusion [13], thus DUO1 has a major role in the specification of functional male gametes. DUO1 is not involved in regulating microspore division and is first expressed in germ cells after asymmetric division. DUO1 is also required for the entry of male germ cells into mitosis and for the germline expression of the G2/M regulator AtCycB1;1. Thus, the germ cell programme under DUO1 control has an important role in regulating core cell cycle machinery specifically in the male germline. The discovery of the dual role of DUO1 as a positive regulator in male germline specification and cell cycle progression is a major advance in uncovering the molecular mechanisms involved in plant sexual reproduction. DUO1 is currently the only regulatory factor that has been shown to be required for gamete specification in plants. Recently we described an independent mechanism for male germ cell cycle regulation where the F-BOX protein FBL17 controls germ cell entry into S-phase via the degradation of the CDKA inhibitors KRP6 and 7 [4]. Taken together these data establish a molecular framework for twin sperm cell production in flowering plants (Figure 5).

Further analysis will shed light on how DUO1 activates its targets, and how DUO1 itself is activated specifically in the male germline. The identification of the role of DUO1 in germ cell specification also provides an exciting platform to develop a detailed regulatory network for male gametogenesis and for comparative studies of the control of sperm cell production. DUO1 homologs are found throughout the land plants from the non-flowering plants *Selaginella moellendorffii* and *Physcomitrella patens* (moss) through to the monocots and dicots (Figure S4). Exploring the functional conservation of DUO1 in different species will reveal if DUO1 has a conserved role in male gamete production, in terms of both of germline mitosis and specification, where DUO1 may regulate the expression of a similar suite of genes such as the conserved GCS1 protein. Such studies may shed light on the evolution of regulatory mechanisms in plant germline development and their significance in double fertilization in flowering plants.

## Materials and Methods

### Plant Material and Transformation

*Arabidopsis* plants were grown at 21°C with a 16 h-light and 8 h-dark cycle or with 24 h light, with variable humidity. Experiments were conducted in the *duo1-1* (in No-0) or the No-0 backgrounds, except for those involving the inducible ectopic expression of mDUO1 and analysis of the DUO1 promoter that were conducted in Col-0. The AtGCS1-AtGCS1::GFP, AtGEX2-GFP and CDG marker lines are also in Col-0. Plants were transformed with *Agrobacterium tumefaciens* (GV3101) using a standard floral dipping method. Transformants were selected either on Murashige and Skoog (MS) agar containing 50 µg/ml kanamycin or 20 µg/ml hygromycin or on soil with 30 µg/ml BASTA (glufosinate ammonium, DHAI PROCIDA) fed by sub-irrigation.

### Vector Construction

Gateway single and multi-site construction (Invitrogen) was used to generate most vectors. DNA was amplified from genomic DNA, cDNA or plasmid DNA by PCR with high fidelity Phusion DNA polymerase (Finnzymes) and primers with suitable *att*achment site (*att*B) adapters (Table S8, *att*B adapters in italics). Full-length *att*B sites were added to each fragment in a second high fidelity PCR. For site-directed mutagenesis of the putative GRSF binding site in the DUO1 promoter a two-step recombinant PCR approach was taken. Two overlapping PCR fragments were generated containing the mutated sequence (underlined in Table S8) and the two fragments joined in a stitching PCR. PCR fragments were cloned into pDONR vectors (Invitrogen; pDONR207 for *AtCycB1;1* cDNA or pDONR221 for *H2B* and *DUO1* and *mDUO1* cDNA, pDONRP4P1R for promoter regions and pDONRP2RP3 for GFP and RFP) via a BP reaction using BP Clonase II (Invitrogen). The product of BP reactions was transformed into alpha-select chemically competent cells (Bioline) and all clones were verified by sequencing.

A multipart LR reaction using LR Clonase plus (Invitrogen) and the destination vector pK7m34GW [32] was used to generate the *AtMGH3* marker, AtMGH3-H2B::GFP. This contains the region upstream of the *AtMGH3* coding region [7] driving expression of a H2B::GFP fusion protein, with the H2B used to give a nuclear GFP signal. The GCS1-GCS1::GFP marker was constructed by inserting a PCR fragment of GFP (primers in Table S8) into an AflII site in the 16^th^ exon of a *Arabidopsis GCS1* genomic DNA fragment in the previously described binary vector [10]. The AtGEX2-GFP marker, with the *GEX2* promoter region driving GFP expression was kindly provided by Shelia McCormick [9].

The vectors DUO1-DUO1::mRFP, DUO1-H2B::mRFP and LAT52-DUO1::mRFP were also generated using gateway multisite cloning and the vectors pK7m34GW or pB7m34GW [32]. DUO1-DUO1::mRFP uses the *DUO1* promoter region to drive expression of a DUO1::mRFP fusion (used to follow the DUO1 protein during pollen development) while DUO1-H2B::GFP uses the *DUO1* promoter to produce a H2B::mRFP fusion protein (used to follow the activity of the DUO1 promoter in *duo1* pollen). The *LAT52* promoter is active in the vegetative cell [23] so was used to ectopically express DUO1::mRFP in the vegetative cell. Vectors to analyse the *DUO1* promoter region were also constructed using gateway multisite cloning.

The *DUO1* mRNA contains a functional recognition site for the microRNA miR159 [21], so for inducible expression of *DUO1* a miR159 resistant version of the *DUO1* cDNA was used containing silent mutations in the miR159 binding site. This was cloned in the vector pMDC7 [33] that contains the XVE estradiol inducible promoter system [22], using a single part LR reaction and LR Clonase II (Invitrogen).

For experiments examining the ability of AtCycB1;1 to complement the *duo1* division phenotype the vectors pB2GW7 and pH2GW7 [34] were modified to contain the *DUO1* and *LAT52* promoters respectively. The 1.2 kb *DUO1* and 609 bp *LAT52* promoter fragments were amplified from cloned sequences using restriction tagged oligonucleotide primer pairs (Table S8). A single part gateway reaction was then used to clone *AtCycB1;1* into the vectors creating DUO1-CycB1;1 and LAT52-CycB1;1. MGH3-CycB1;1::GFP was generated using a multipart gateway reaction.

### Inducible Expression of DUO1

T2 seed from transgenic Col-O plants were grown in MSO plates containing 20 µg/ml hygromycin in standard conditions for 12 days. 25 seedlings were transferred to either control plants containing 0.002% v/v DMSO or induction plates contain 2 µM 17β-estradiol dissolved in DMSO. Plants were returned to the growth room for a further 24 h, before being snap frozen in liquid nitrogen.

### RT-PCR Analysis

Pollen from ecotype Landsberg *erecta* at different stages of development was isolated and RNA extracted as described [31]. For RT-PCR on seedling ectopically expressing *mDUO1* and for *DUO1* promoter analysis, RNA was extracted from frozen samples using the Qiagen RNeasy Kit. Samples of 750 ng or 1 µg of total RNA for pollen stages and seedlings, respectively, were reverse transcribed in a 20 µl reaction using Superscript II RNase H reverse transcriptase (Invitrogen) and an oligodT primer as per the manufactures instructions. For PCR amplification 1 µl of a 10× (pollen stages) or 5× (seedling) diluted cDNA was used in a 25 µl reaction using Biotaq DNA polymerase (Bioline) and 12.5 pmol of each primer (Table S9). PCR conditions were: 96°C for 1 min, 30 to 40 cycles at 96°C for 30 s, 55°C for 30 s, 72°C for 40 s followed by 5 min at 72°C. Histone H3 (At4g40040) was used as a control.

### Analysis and Imaging of Pollen

Mature pollen was stained with DAPI (4′-6-Diamidino-2-phenylindole) as described previously [35]. Staining for GUS activity was performed as described [36] with inflorescences incubated in GUS buffer (100 mM sodium-phosphate, pH 7; 5 mM EDTA, 0.1% Triton X-100) with 1 mM X-gluc (5-bromo-4-chloro-3-indolyl b-D-glucuronide) and 0.5 mM K_3_Fe[CN]_6_, at 37°C for 1–3 days. Stained inflorescences were then cleared with 70% ethanol. Pollen was dissected out and stained with 0.8 µg/ml DAPI in GUS buffer. Phenotypic analysis of pollen was conducted on a Nikon TE2000-E inverted microscope (Nikon, Japan). Bright field and DIC images were captured with a Nikon-D100 camera (Model MH-18, Japan) and fluorescence images were captured with HAMAMATSU – ORCA-ER digital camera (Model C4742-95, Japan) using Openlab software version 5.0.2. (Improvision).

For confocal laser scanning microscopy (CLSM) pollen from buds at different stages of development was teased out of the anther with a needle and mounted in 0.3 M mannitol and mature pollen was released directly into 0.3 M mannitol. Pollen was viewed with a Nikon TE2000-E inverted microscope and C1 confocal system using Melles Griot Argon Ion (emission 488 nm) and Melles Griot Helium-Neon (emission 543 nm) lasers, detection filters for GFP and RFP, and EZ-C1 control and imaging software.

## Supporting Information

Figure S1 Male germline development in *Arabidopsis*. Following male meiosis a tetrad of haploid microspores is produced surrounded by a thick callose wall (yellow). Individual microspores released by dissolution of the callose wall undergo two mitotic divisions to produce mature tricellular pollen grains. The first asymmetric division gives rise to a vegetative cell (blue) that will form the pollen tube and a smaller male germ cell (pink) that divides within the vegetative cell cytoplasm to form twin sperm cells (red). Cell cycle progression in the male germ lineage is illustrated below.
(0.14 MB DOC)

Figure S2 Expression of *AtCycB1;1* in developing pollen. RT-PCR analysis of *AtCycB1;1* expression in uninucleate microspores (UNM), bicellular pollen (BCP), tricellular pollen (TCP) and mature pollen (MPG). Histone H3 was used as a control, gDNA, genomic DNA.
(0.08 MB DOC)

Figure S3 Viability of LAT52-DUO1::RFP pollen. (A,B) Hemizygous LAT52-DUO1::RFP pollen population showing cosegregation of aberrant cell morphology (A) and ectopic expression of MGH3-H2B::GFP (B). Arrows indicate aberrant pollen. (C,D) Ultrastructure of wild type (C) and aberrant pollen (D) in mature anthers of plants segregating for LAT52-DUO1::RFP expression. (E,F) Hemizygous LAT52-DUO1::RFP pollen population showing cosegregation of negative FDA staining (E) and aberrant cell morphology (F).
(0.94 MB DOC)

Figure S4 Alignment of DUO1 homologs from land plants. The *Arabidopsis* DUO1 protein was used in BLAST searches of databases through NCBI, TIGR plant genomes and JGI Eukaryotic genomes to identify DUO1 homologs. Sequences were aligned with CLUSTALW using default settings. DUO1 proteins are characterized by a supplementary lysine (K^66^ in AtDUO1) which is never observed in other plant MYB sequences [20], indicated by * above the sequence. The two MYB domains are indicated by a blue (R2) and red (R3) line under the sequence. Species: At = *Arabidopsis thaliana*, Rc = *Ricinus communis* (Castor bean), Pt = *Populus trichocarpa* (Poplar), Nt, *Nicotiana tabacum* (Tobacco), Os = *Oryza sativa* (Rice), Ll = *Lilium longiflorum* (Lily), Sm = *Selaginella moellendorffii* and Pp = *Physcomitrella patens* (moss). There are two DUO1-related proteins, named A and B, in *P. patens* and *S. moellendorffii*.
(1.61 MB DOC)

Table S1 Expression of male germline marker constructs in wild type, *cdka* and *duo1* pollen. Mature pollen from heterozygous *cdka* and *duo1* plants that were homozygous for individual marker constructs was stained with DAPI and observed by fluorescence microscopy. The phenotype of each pollen grain was determined and the presence (+) or absence (−) of GFP or RFP in the germline scored.
(0.03 MB DOC)

Table S2 Analysis of LAT52-DUO1::RFP pollen. Mature pollen from plants homozygous for MGH3-H2B::GFP and heterozygous for LAT52-DUO1::RFP (three separate T1 lines, A1–A3) was analysed by fluorescence microscopy for GFP and RFP expression. Control plants homozygous for MGH3-H2B::GFP show 100% sperm cell-specific GFP signal (SC GFP). Approximately 50% of pollen from each hemizygous LAT52-DUO1::RFP line showed GFP signal in the vegetative nucleus (VN GFP). RFP was also detected in the vegetative nucleus (VN RFP) of these lines, although its detection levels varied between individual lines. Data for each marker is presented as a percentage, with the number of pollen grains indicated in parentheses.
(0.03 MB DOC)

Table S3 Complementation of *duo1* pollen by AtCycB1;1. DAPI stained pollen from heterozygous *duo1* individuals either not transformed, transformed with AtMGH3-AtCycB1;1::GFP (a control not expressed in *duo1* pollen, see Figure 1), LAT52-AtCycB1;1 (a control expressed in the vegetative cell but not the germline) or DUO1-AtCycB1:1 were counted to analyse the proportion of tricellular and bicellular (*duo1*) pollen. The Chi-square test was applied to determine if the ratio of wild type to mutant pollen was significantly different from the expected 1∶1 ratio (ns = not significantly different (p<0.05); * = significantly different (p<0.05).)
(0.12 MB DOC)

Table S4 Marker expression in *duo1* pollen complemented with DUO1-AtCycB1;1. Pollen from plants homozygous for AtMGH3-H2B::GFP (AtMGH3) or AtGCS1-AtGCS1::GFP (AtGCS1) without the DUO1-AtCycB1;1 construct (control) or showing partial complementation by DUO1-AtCycB1;1 was stained with DAPI and observed by fluorescence microscopy. The phenotype of each pollen grain was determined and the presence (+) or absence (−) of GFP in the germline scored.
(0.03 MB DOC)

Table S5 Transmission of the *duo1* allele after introduction of DUO1-AtCycB1:1. When *duo1* heterozygotes are selfed the F1 progeny display a 1∶1 ratio of WT to *duo1* plants. A similar ratio is observed when *duo1* heterozygotes partially complemented by DUO1-AtCycB1;1 (+/*duo1DC*) are selfed. The *duo1* allele is not transmitted through the male in either heterozygous *duo1* or *duo1*-complemented plants (+/*duo1DC*). The DUO1-AtCycB1;1 transgene (pptR) is transmitted as a single locus in selfed individuals and normally through the male when crossed to wild type female. TE^male^ represents the transmission efficiency of *duo1* through pollen (mutant/wild type X 100); na = not applicable.
(0.03 MB DOC)

Table S6 Aberrant morphology of pollen containing LAT52-DUO1::RFP. GFP and RFP signals along with cell morphology were analysed for each pollen grain from line A3 (Table S2), that was homozygous for MGH3-H2B::GFP and hemizygous for LAT52-DUO1::RFP. Approximately 50% of pollen possessed aberrant morphology (see Figure S3), and of these pollen grains all were positive for vegetative nucleus GFP (+ VN GFP), indicating the presence of LAT52-DUO1::RFP. Data is presented as numbers of pollen grains scored from the population.
(0.03 MB DOC)

Table S7 Viability of hemizygous LAT52-DUO1::RFP pollen. Viability of mature pollen from plants homozygous for MGH3-H2B::GFP and heterozygous for LAT52-DUO1::RFP (three separate T1 lines, A1–A3) was analysed by fluorescence microscopy after FDA staining. Pollen from control plants homozygous for MGH3-H2B::GFP is almost all viable. Pollen viability is reduced by up to 50% in hemizygous LAT52-DUO1::RFP lines. Data for each marker is presented as a percentage, with the number of pollen grains counted indicated in parentheses.
(0.03 MB DOC)

Table S8 Primers used for vector construction.
(0.05 MB DOC)

Table S9 Primers used in RT-PCR analyses.
(0.03 MB DOC)
