# Supplementary figures and images for: A Plant Germline-Specific Integrator of Sperm Specification and Cell Cycle Progression
Source: PLoS Genet. 2009 Mar 20;5(3):e1000430. doi: 10.1371/journal.pgen.1000430 (PMC2653642; doi:10.1371/journal.pgen.1000430)

**
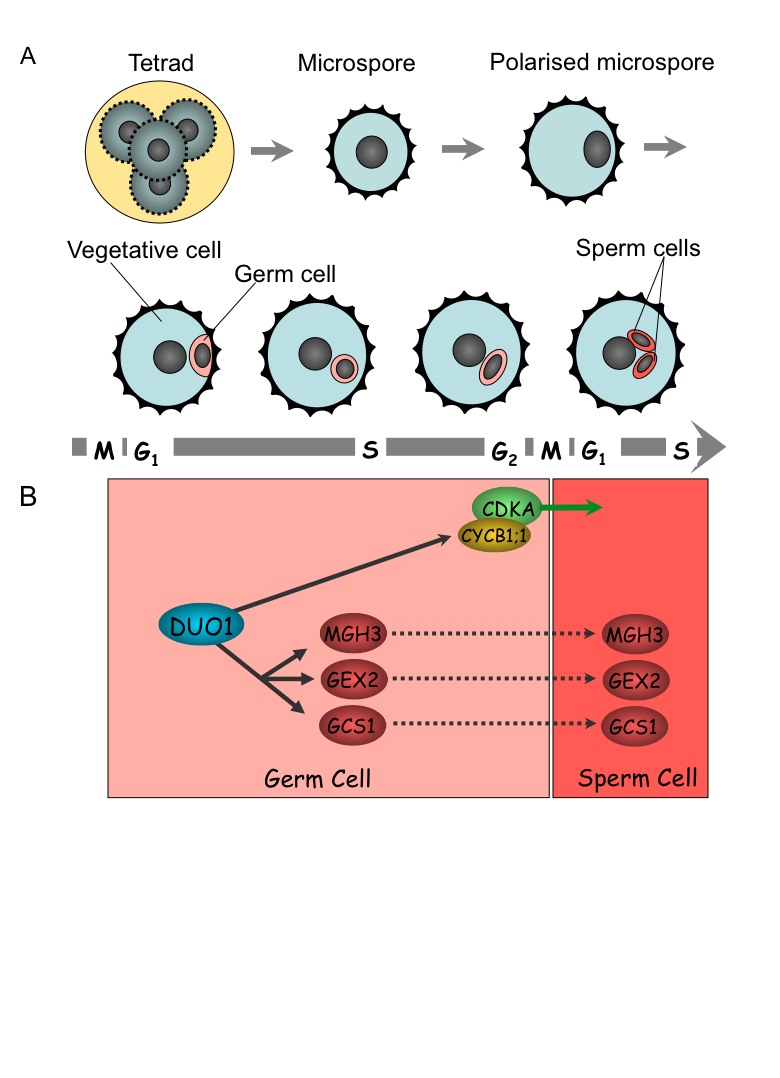
**

Supplement: Figure S1 — Male germline development in Arabidopsis. Following male meiosis a tetrad of haploid microspores is produced surrounded by a thick callose wall (yellow). Individual microspores released by dissolution of the callose wall undergo two mitotic divisions to produce mature tricellular pollen grains. The first asymmetric division gives rise to a vegetative cell (blue) that will form the pollen tube and a smaller male germ cell (pink) that divides within the vegetative cell cytoplasm to form twin sperm cells (red). Cell cycle progression in the male germ lineage is illustrated below. (0.14 MB DOC) [file pgen.1000430.s001.doc]

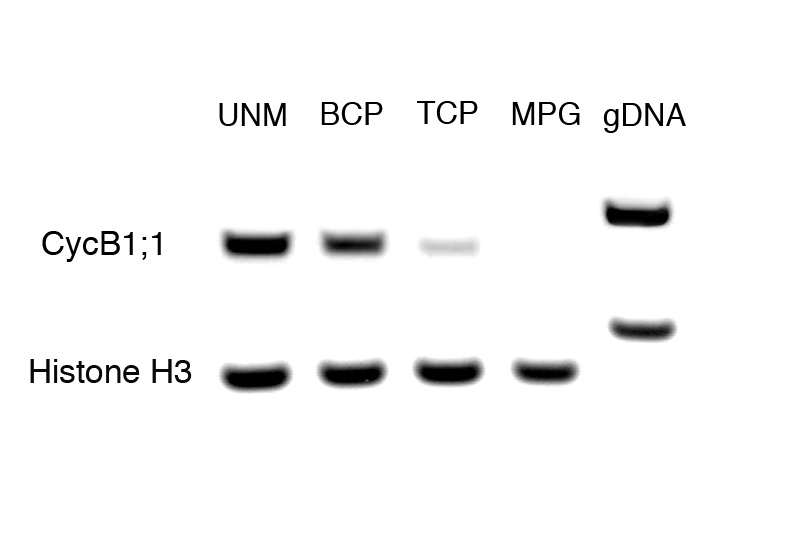

Supplement: Figure S2 — Expression of AtCycB1;1 in developing pollen. RT-PCR analysis of AtCycB1;1 expression in uninucleate microspores (UNM), bicellular pollen (BCP), tricellular pollen (TCP) and mature pollen (MPG). Histone H3 was used as a control, gDNA, genomic DNA. (0.08 MB DOC) [file pgen.1000430.s002.doc]

**
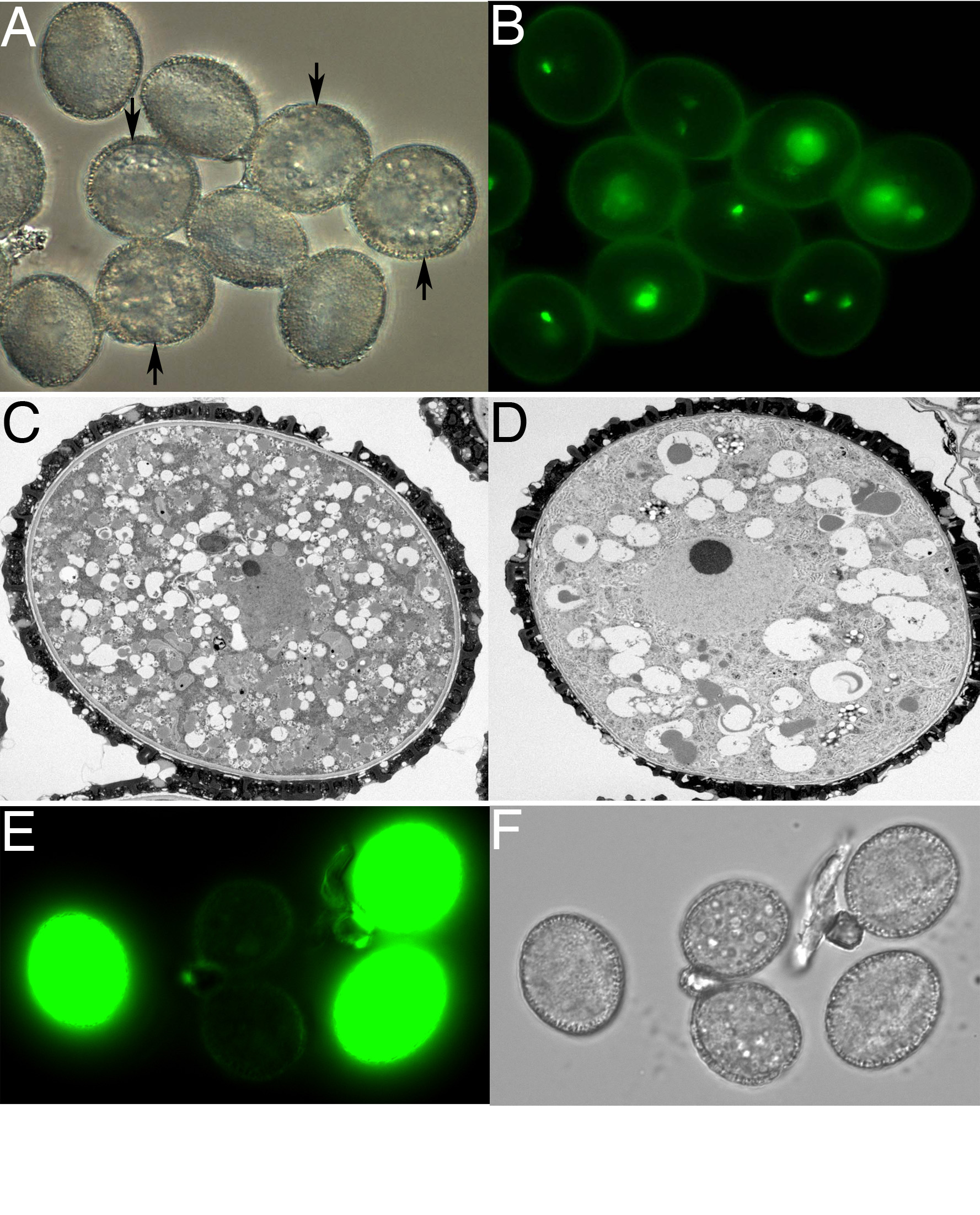
**

Supplement: Figure S3 — Viability of LAT52-DUO1::RFP pollen. (A,B) Hemizygous LAT52-DUO1::RFP pollen population showing cosegregation of aberrant cell morphology (A) and ectopic expression of MGH3-H2B::GFP (B). Arrows indicate aberrant pollen. (C,D) Ultrastructure of wild type (C) and aberrant pollen (D) in mature anthers of plants segregating for LAT52-DUO1::RFP expression. (E,F) Hemizygous LAT52-DUO1::RFP pollen population showing cosegregation of negative FDA staining (E) and aberrant cell morphology (F). (0.94 MB DOC) [file pgen.1000430.s003.doc]

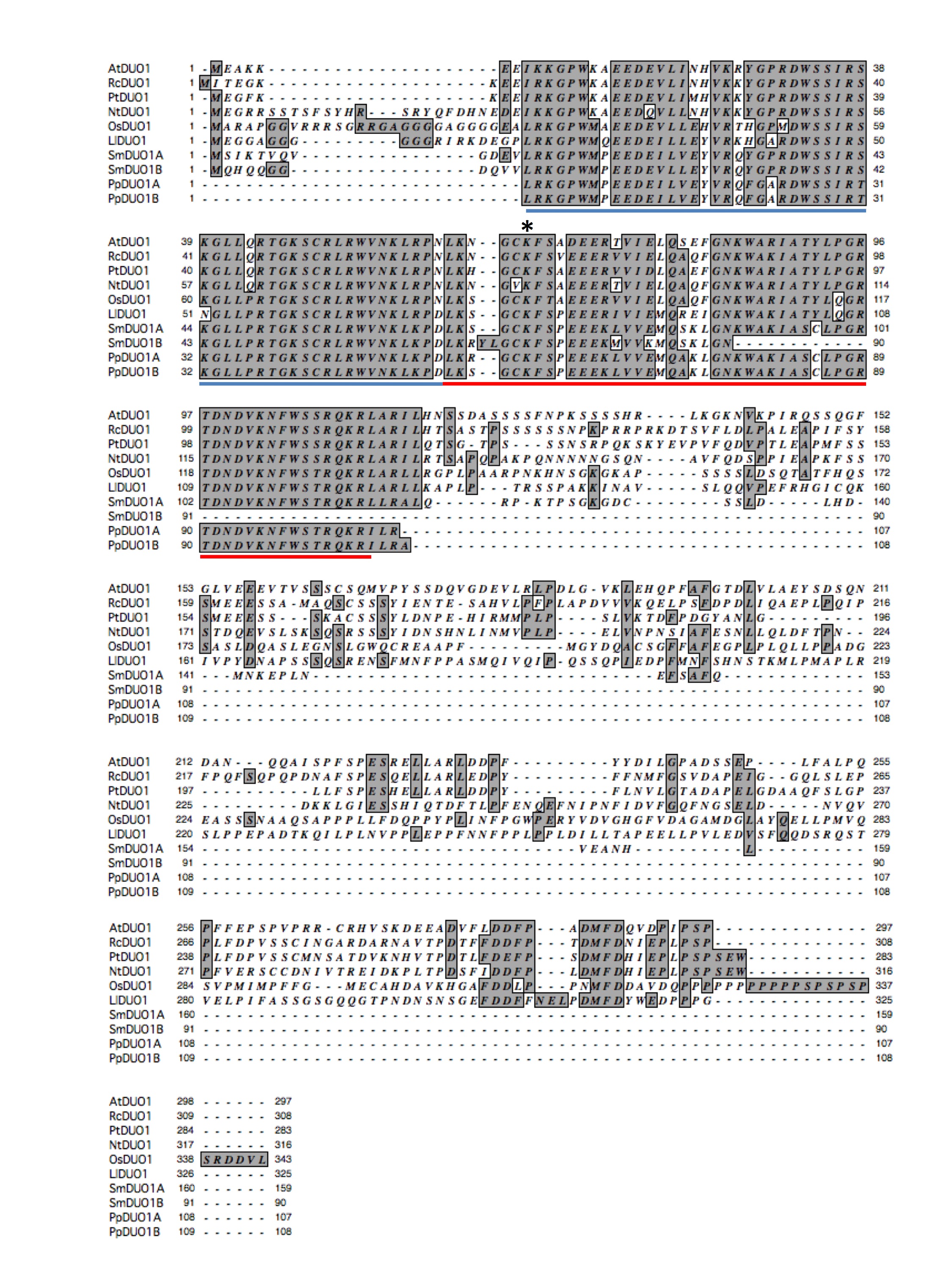

Supplement: Figure S4 — Alignment of DUO1 homologs from land plants. The Arabidopsis DUO1 protein was used in BLAST searches of databases through NCBI, TIGR plant genomes and JGI Eukaryotic genomes to identify DUO1 homologs. Sequences were aligned with CLUSTALW using default settings. DUO1 proteins are characterized by a supplementary lysine (K66 in AtDUO1) which is never observed in other plant MYB sequences [20], indicated by * above the sequence. The two MYB domains are indicated by a blue (R2) and red (R3) line under the sequence. Species: At = Arabidopsis thaliana, Rc = Ricinus communis (Castor bean), Pt = Populus trichocarpa (Poplar), Nt, Nicotiana tabacum (Tobacco), Os = Oryza sativa (Rice), Ll = Lilium longiflorum (Lily), Sm = Selaginella moellendorffii and Pp = Physcomitrella patens (moss). There are two DUO1-related proteins, named A and B, in P. patens and S. moellendorffii. (1.61 MB DOC) [file pgen.1000430.s004.doc]
