# Supplementary material for: A Plant Germline-Specific Integrator of Sperm Specification and Cell Cycle Progression
Source: PLoS Genet. 2009 Mar 20;5(3):e1000430. doi: 10.1371/journal.pgen.1000430 (PMC2653642; doi:10.1371/journal.pgen.1000430)
Supplement: Table S1 — Expression of male germline marker constructs in wild type, cdka and duo1 pollen. Mature pollen from heterozygous cdka and duo1 plants that were homozygous for individual marker constructs was stained with DAPI and observed by fluorescence microscopy. The phenotype of each pollen grain was determined and the presence (+) or absence (−) of GFP or RFP in the germline scored. (0.03 MB DOC) [file pgen.1000430.s005.doc]

| **Marker** | **+ GFP/RFP** | | | **- GFP/RFP** | | |
| --- | --- | --- | --- | --- | --- | --- |
|  | **WT** | ***cdka*** | ***duo1*** | **WT** | ***cdka*** | ***duo1*** |
| AtMGH3-H2B::GFP | 776 | 232 | 0 | 0 | 0 | 280 |
| AtGEX2::GFP | 756 | 154 | 69 | 14 | 0 | 398 |
| AtGCS1-AtGCS1::GFP | 795 | 235 | 5 | 6 | 0 | 317 |
| DUO1-H2B::RFP | 988 | 231 | 529 | 10 | 0 | 7 |
