# Supplementary material for: A Plant Germline-Specific Integrator of Sperm Specification and Cell Cycle Progression
Source: PLoS Genet. 2009 Mar 20;5(3):e1000430. doi: 10.1371/journal.pgen.1000430 (PMC2653642; doi:10.1371/journal.pgen.1000430)
Supplement: Table S2 — Analysis of LAT52-DUO1::RFP pollen. Mature pollen from plants homozygous for MGH3-H2B::GFP and heterozygous for LAT52-DUO1::RFP (three separate T1 lines, A1–A3) was analysed by fluorescence microscopy for GFP and RFP expression. Control plants homozygous for MGH3-H2B::GFP show 100% sperm cell-specific GFP signal (SC GFP). Approximately 50% of pollen from each hemizygous LAT52-DUO1::RFP line showed GFP signal in the vegetative nucleus (VN GFP). RFP was also detected in the vegetative nucleus (VN RFP) of these lines, although its detection levels varied between individual lines. Data for each marker is presented as a percentage, with the number of pollen grains indicated in parentheses. (0.03 MB DOC) [file pgen.1000430.s006.doc]

| **Line** | **% SC GFP** | **% VN GFP** | **% VN RFP** |
| --- | --- | --- | --- |
| Control | 100 (320) | 0 (320) | 0 (320) |
| A1 | 100 (102) | 46 (102) | 10 (103) |
| A2 | 100 (102) | 50 (102) | 36 (117) |
| A3 | 100 (448) | 49 (448) | 24 (245) |
