# Supplementary material for: A Plant Germline-Specific Integrator of Sperm Specification and Cell Cycle Progression
Source: PLoS Genet. 2009 Mar 20;5(3):e1000430. doi: 10.1371/journal.pgen.1000430 (PMC2653642; doi:10.1371/journal.pgen.1000430)
Supplement: Table S3 — Complementation of duo1 pollen by AtCycB1;1. DAPI stained pollen from heterozygous duo1 individuals either not transformed, transformed with AtMGH3-AtCycB1;1::GFP (a control not expressed in duo1 pollen, see Figure 1), LAT52-AtCycB1;1 (a control expressed in the vegetative cell but not the germline) or DUO1-AtCycB1:1 were counted to analyse the proportion of tricellular and bicellular (duo1) pollen. The Chi-square test was applied to determine if the ratio of wild type to mutant pollen was significantly different from the expected 1∶1 ratio (ns = not significantly different (p<0.05); * = significantly different (p<0.05).) (0.12 MB DOC) [file pgen.1000430.s007.doc]

| **Construct** | **Tricellular** | **Bicellular** | **% Tricellular** | **% Bicellular** | **Significance** |
| --- | --- | --- | --- | --- | --- |
| Not transformed | 100 | 99 | 50.3 | 49.7 | ns |
|  | 316 | 294 | 51.8 | 48.2 | ns |
|  | 221 | 227 | 49.3 | 50.7 | ns |
| AtMGH3-AtCycB1;1::GFP | 207 | 205 | 50.2 | 49.8 | ns |
|  | 214 | 207 | 50.8 | 49.2 | ns |
|  | 203 | 189 | 51.8 | 48.2 | ns |
|  | 221 | 224 | 49.7 | 50.3 | ns |
|  | 201 | 216 | 48.2 | 51.8 | ns |
|  | 212 | 210 | 50.2 | 49.8 | ns |
|  | 257 | 209 | 55.2 | 44.8 | ns |
|  | 225 | 212 | 51.5 | 48.5 | ns |
|  | 221 | 213 | 50.9 | 49.1 | ns |
|  | 215 | 217 | 49.8 | 50.2 | ns |
|  | 201 | 177 | 53.2 | 46.8 | ns |
|  | 201 | 213 | 48.6 | 51.4 | ns |
|  | 203 | 222 | 47.8 | 52.2 | ns |
|  | 206 | 198 | 51.0 | 49.0 | ns |
|  | 219 | 189 | 53.7 | 46.3 | ns |
|  | 232 | 211 | 52.4 | 47.6 | ns |
|  | 219 | 231 | 48.7 | 51.3 | ns |
| LAT52-AtCycB1;1 | 276 | 264 | 51.1 | 48.9 | ns |
|  | 280 | 256 | 52.2 | 47.8 | ns |
|  | 320 | 308 | 51.0 | 49.0 | ns |
|  | 180 | 195 | 48.0 | 52.0 | ns |
|  | 214 | 172 | 55.4 | 44.6 | ns |
|  | 141 | 136 | 50.9 | 49.1 | ns |
|  | 240 | 236 | 50.4 | 49.6 | ns |
|  | 217 | 209 | 50.9 | 49.1 | ns |
|  | 211 | 215 | 49.5 | 50.5 | ns |
|  | 255 | 207 | 55.2 | 44.8 | ns |
|  | 212 | 210 | 50.2 | 49.8 | ns |
|  | 215 | 190 | 53.1 | 46.9 | ns |
|  | 221 | 229 | 49.1 | 50.9 | ns |
|  | 221 | 233 | 48.7 | 51.3 | ns |
|  | 227 | 214 | 51.5 | 48.5 | ns |
|  | 205 | 191 | 51.8 | 48.2 | ns |

Table S3 continued

| **Construct** | **Tricellular** | **Bicellular** | **% Tricellular** | **% Bicellular** | **Significance** |
| --- | --- | --- | --- | --- | --- |
| DUO1-AtCycB1;1 | 653 | 416 | 61.1 | 38.9 | * |
|  | 197 | 136 | 59.2 | 40.8 | * |
|  | 90 | 77 | 53.9 | 46.1 | ns |
|  | 652 | 405 | 61.7 | 38.3 | * |
|  | 142 | 88 | 61.7 | 38.3 | * |
|  | 204 | 148 | 58.0 | 42.0 | * |
|  | 207 | 145 | 58.8 | 41.2 | * |
|  | 237 | 171 | 58.1 | 41.9 | * |
|  | 223 | 159 | 58.4 | 41.6 | * |
|  | 192 | 158 | 54.9 | 45.1 | ns |
|  | 182 | 154 | 54.2 | 45.8 | ns |
|  | 252 | 184 | 57.8 | 42.2 | * |
|  | 1412 | 973 | 59.2 | 40.8 | * |
|  | 142 | 130 | 52.2 | 47.8 | ns |
|  | 212 | 180 | 54.1 | 45.9 | ns |
|  | 733 | 379 | 65.9 | 34.1 | * |
|  | 121 | 100 | 54.8 | 45.2 | ns |
|  | 221 | 118 | 65.2 | 34.8 | * |
|  | 326 | 197 | 62.3 | 37.7 | * |
|  | 213 | 138 | 60.7 | 39.3 | * |
|  | 241 | 164 | 59.5 | 40.5 | * |
|  | 275 | 169 | 61.9 | 38.1 | * |
|  | 210 | 113 | 65.0 | 35.0 | * |
|  | 224 | 165 | 57.6 | 42.4 | * |
|  | 348 | 192 | 64.4 | 35.6 | * |
|  | 293 | 147 | 66.6 | 33.4 | * |
|  | 271 | 140 | 65.9 | 34.1 | * |
|  | 222 | 191 | 53.8 | 46.2 | ns |
|  | 234 | 190 | 55.2 | 44.8 | ns |
|  | 224 | 133 | 62.7 | 37.3 | * |
|  | 207 | 197 | 51.2 | 48.8 | ns |
|  | 262 | 199 | 56.8 | 43.2 | * |
|  | 230 | 195 | 54.1 | 45.9 | ns |
|  | 257 | 194 | 57.0 | 43.0 | * |
|  | 204 | 179 | 53.3 | 46.7 | ns |
|  | 246 | 186 | 56.9 | 43.1 | * |
|  | 206 | 188 | 52.3 | 47.7 | ns |
|  | 200 | 176 | 53.2 | 46.8 | ns |
|  | 206 | 232 | 47.0 | 53.0 | ns |
|  | 245 | 171 | 58.9 | 41.1 | * |
|  | 219 | 209 | 51.2 | 48.8 | ns |
|  | 253 | 169 | 60.0 | 40.0 | * |

Table S3 continued

| **Construct** | **Tricellular** | **Bicellular** | **% Tricellular** | **% Bicellular** | **Significance** |
| --- | --- | --- | --- | --- | --- |
| DUO1-AtCycB1;1 | 220 | 211 | 51.0 | 49.0 | ns |
|  | 205 | 204 | 50.1 | 49.9 | ns |
|  | 238 | 168 | 58.6 | 41.4 | * |
|  | 202 | 205 | 49.6 | 50.4 | ns |
|  | 237 | 178 | 57.1 | 42.9 | * |
|  | 246 | 185 | 57.1 | 42.9 | * |
|  | 223 | 142 | 61.1 | 38.9 | * |
