# Supplementary material for: A Plant Germline-Specific Integrator of Sperm Specification and Cell Cycle Progression
Source: PLoS Genet. 2009 Mar 20;5(3):e1000430. doi: 10.1371/journal.pgen.1000430 (PMC2653642; doi:10.1371/journal.pgen.1000430)
Supplement: Table S4 — Marker expression in duo1 pollen complemented with DUO1-AtCycB1;1. Pollen from plants homozygous for AtMGH3-H2B::GFP (AtMGH3) or AtGCS1-AtGCS1::GFP (AtGCS1) without the DUO1-AtCycB1;1 construct (control) or showing partial complementation by DUO1-AtCycB1;1 was stained with DAPI and observed by fluorescence microscopy. The phenotype of each pollen grain was determined and the presence (+) or absence (−) of GFP in the germline scored. (0.03 MB DOC) [file pgen.1000430.s008.doc]

|  |  | **Tricellular** | | **Bicellular** | |
| --- | --- | --- | --- | --- | --- |
| **marker** | **construct** | **+ GFP** | **- GFP** | **+ GFP** | **- GFP** |
| AtMGH3 | control | 498 | 1 | 0 | 454 |
| AtGCS1 | control | 602 | 10 | 5 | 571 |
| AtMGH3 | DUO1-AtCycB1;1 | 462 | 92 | 0 | 360 |
| AtGCS1 | DUO1-AtCycB1;1 | 502 | 99 | 0 | 386 |
