# Supplementary material for: A Plant Germline-Specific Integrator of Sperm Specification and Cell Cycle Progression
Source: PLoS Genet. 2009 Mar 20;5(3):e1000430. doi: 10.1371/journal.pgen.1000430 (PMC2653642; doi:10.1371/journal.pgen.1000430)
Supplement: Table S5 — Transmission of the duo1 allele after introduction of DUO1-AtCycB1:1. When duo1 heterozygotes are selfed the F1 progeny display a 1∶1 ratio of WT to duo1 plants. A similar ratio is observed when duo1 heterozygotes partially complemented by DUO1-AtCycB1;1 (+/duo1DC) are selfed. The duo1 allele is not transmitted through the male in either heterozygous duo1 or duo1-complemented plants (+/duo1DC). The DUO1-AtCycB1;1 transgene (pptR) is transmitted as a single locus in selfed individuals and normally through the male when crossed to wild type female. TEmale represents the transmission efficiency of duo1 through pollen (mutant/wild type X 100); na = not applicable. (0.03 MB DOC) [file pgen.1000430.s009.doc]

| **Female** | **Male** | **F1 phenotype** | | **TE^male^** | **pptR:pptS** | **Ratio** |
| --- | --- | --- | --- | --- | --- | --- |
|  |  | **WT** | **+/*duo1*** |  |  |  |
| +/*duo1* | +/*duo1** | 1912 | 1819 | na | na | na |
| **+/***duo1DC^a^* | **+/***duo1DC^a^* | 12 | 12 | na | 151:44 | 3:1 |
| **+/***duo1DC^b^* | **+/***duo1DC^b^* | 14 | 10 | na | 241:82 | 3:1 |
| **+/+** | +/*duo1** | 530 | 0 | 0 | na | na |
| **+/+** | **+/***duo1DC^a^* | 34 | 0 | 0 | 16:18 | 1:1 |
| **+/+** | **+/***duo1DC^b^* | 77 | 0 | 0 | 37:40 | 1:1 |

* data from [24]

^a,b^ data from two independent transformed lines
