# Supplementary material for: A Plant Germline-Specific Integrator of Sperm Specification and Cell Cycle Progression
Source: PLoS Genet. 2009 Mar 20;5(3):e1000430. doi: 10.1371/journal.pgen.1000430 (PMC2653642; doi:10.1371/journal.pgen.1000430)
Supplement: Table S6 — Aberrant morphology of pollen containing LAT52-DUO1::RFP. GFP and RFP signals along with cell morphology were analysed for each pollen grain from line A3 (Table S2), that was homozygous for MGH3-H2B::GFP and hemizygous for LAT52-DUO1::RFP. Approximately 50% of pollen possessed aberrant morphology (see Figure S3), and of these pollen grains all were positive for vegetative nucleus GFP (+ VN GFP), indicating the presence of LAT52-DUO1::RFP. Data is presented as numbers of pollen grains scored from the population. (0.03 MB DOC) [file pgen.1000430.s010.doc]

| **Morphology** | **+VN GFP**  **+VN RFP** | **+VN GFP**  **-VN RFP** | **-VN GFP**  **+VN RFP** | **-VN GFP**  **-VN RFP** |
| --- | --- | --- | --- | --- |
| Aberrant | 153 | 83 | 0 | 0 |
| Wild type | 0 | 3 | 0 | 238 |
