# Supplementary material for: A Plant Germline-Specific Integrator of Sperm Specification and Cell Cycle Progression
Source: PLoS Genet. 2009 Mar 20;5(3):e1000430. doi: 10.1371/journal.pgen.1000430 (PMC2653642; doi:10.1371/journal.pgen.1000430)
Supplement: Table S7 — Viability of hemizygous LAT52-DUO1::RFP pollen. Viability of mature pollen from plants homozygous for MGH3-H2B::GFP and heterozygous for LAT52-DUO1::RFP (three separate T1 lines, A1–A3) was analysed by fluorescence microscopy after FDA staining. Pollen from control plants homozygous for MGH3-H2B::GFP is almost all viable. Pollen viability is reduced by up to 50% in hemizygous LAT52-DUO1::RFP lines. Data for each marker is presented as a percentage, with the number of pollen grains counted indicated in parentheses. (0.03 MB DOC) [file pgen.1000430.s011.doc]

| **Line** | **% FDA staining** |
| --- | --- |
| Control | 98 (119) |
| A1 | 68 (63) |
| A2 | 56 (118) |
| A3 | 51 (119) |
