# Supplementary material for: A Plant Germline-Specific Integrator of Sperm Specification and Cell Cycle Progression
Source: PLoS Genet. 2009 Mar 20;5(3):e1000430. doi: 10.1371/journal.pgen.1000430 (PMC2653642; doi:10.1371/journal.pgen.1000430)
Supplement: Table S8 — Primers used for vector construction. (0.05 MB DOC) [file pgen.1000430.s012.doc]

| **Name** | **Sequence (5’ to 3’)** | **Use** |
| --- | --- | --- |
| CycB1;1cDNAF | *AAAAAAGCAGGCT*TCATGATGACTTCTCGTTCGATTGTTC | AtCycB1;1 cDNA |
| CycB1;1cDNAR | *CAAGAAAGCTGGGT*CCTAAGCAGATTCAGTTCCGGTCAAC |
| DUO1cDNAattB1F | *AAAAAGCAGGCTC*TATGGAAGCGAAGAAGGAAG | DUO1 cDNA |
| DUO1cDNAattB2R | *AGAAAGCTGGGT*AAGGACTTGGGATTGGATCAAC |
| DUO1attB4F | *TGTATAGAAAAGTTG*ACGTCCGAAGTTTCCCTCTTGG | DUO1 promoter |
| DUO1attB1R | *TTTTGTACAAACTTG*CGCTAATCGATCTCTCTCTCG |
| MGH3attB4F | *TGTATAGAAAAGTTG*TGTTGGCCTATCACGTTGAA | AtMGH3 promoter |
| MGH3attB1R | *TTTTGTACAAACTTG*TTCTTCGAGAGAACGATGATG |
| LAT52attB4F | *TGTATAGAAAAGTTG*TTGAGGAATGATCGATTCTGG | LAT52 promoter |
| LAT52attB1R | *TTTTGTACAAACTTG*GAAATTTTTTTTTTGGTGTGTG |
| H2Batt1F | *AAAAAGCAGGCT*CAATGGCGAAGGCAGATAAGAAACC | H2B cDNA |
| H2Batt2R | *AGAAAGCTGGGT*CCCAGCTCCAGCAGAACTCGTAAAC |
| mGFP6attB2F | *TCTTGTACAAAGTGG*AAATGAGTAAAGGAGAAGAACTT | mGFP6 HIS tag CDS |
| mGFPHIS6attB3R | *TGTATAATAAAGTTG*TTAGTGGTGGTGGTGGTGGTG |
| mRFPattB2F | *TCTTGTACAAAGTGG*CGATGGCCTCCTCCGAGGACG | mRFP CDS |
| mRFPattB3R | *TGTATAATAAAGTTG*TTAGGCGCCGGTGGAGTGG |
| DUO1F SacI | AGAA**GAGCTC**GTCCGAAGTTTCCCTCTTGG | Construction of pBDUO1GW7 |
| DUO1R SpeI | TTTT**ACTAGT**CGCTAATCGATCTCTCTCTCG |
| LAT52F SacI | TCAG**GAGCTC**TTGAGGAATG ATCGATTCTGG | Construction of pHLAT52GW7 |
| LAT52R SpeI | CCAT**ACTAGT**GAATTTTTTTTTTGGTGTGTG |
| sGFPF | TGAGG**CTTAAG**CATGGTGAGCAAGGGCGAGGA | AtGCS1-AtGSC1::GFP |
| sGFPR | TGAGG**CTTAAG**TTGTACAGCTCGTCCATGCCG |
| DUO1msF | CTATCTCATGACGGACTGAGTTTGTCTGGCCTTTG | DUO1 promoter with mutated GRSF binding site |
| DUO1msR | GATTGCTCTCATGAAGATTTCACCAGATTTGAAC |
| DUO1-723attB4F | *TGTATAGAAAAGTTG*GTGGATTCGGAGCCATATCA | DUO1 promoter analysis |
| DUO1-412attB4F | *TGTATAGAAAAGTTG*TGGTTTGAGTCAACCGTCTTT | DUO1 promoter analysis |
| DUO1-363attB4F | *TGTATAGAAAAGTTG*AAAGAATTCAATGTGCAAAGTC | DUO1 promoter analysis |
| DUO1-155attB4F | *TGTATAGAAAAGTTG*CGAGTCTCGAAGATCCTTCAC | DUO1 promoter analysis |
| DUO1-18attB4F | *TGTATAGAAAAGTTG*GGGGAGTGGGGTACTGAAAG | DUO1 promoter analysis |

BOLD – restriction enzyme recognition sequence

Italics – *att*B adapter sequence

Underline – mutated putative GRSF binding site
