# Supplementary material for: A Plant Germline-Specific Integrator of Sperm Specification and Cell Cycle Progression
Source: PLoS Genet. 2009 Mar 20;5(3):e1000430. doi: 10.1371/journal.pgen.1000430 (PMC2653642; doi:10.1371/journal.pgen.1000430)
Supplement: Table S9 — Primers used in RT-PCR analyses. (0.03 MB DOC) [file pgen.1000430.s013.doc]

| **Name** | **Sequence (5’ to 3’)** |
| --- | --- |
| AtCycB1;1RT-F | AAGAGAAACGCAGTACCAAAGC |
| AtCycB1;1RT-R | ATATTCCACAGCTGCGAGGT |
| MGH3RTF | GAGTCAAGAGGGCTCACCGT |
| MGH3RTR | ATACAAATTCACAGAACCATCATGATG |
| GEX2RTF | CAAACATTGAATGGTGGTCC |
| GEX2RTR | ATGGCATACTAGAGATGCTC |
| GCS1RTF | TGGTTTAGTCGATTTCATCACC |
| GCS1RTR | TACGTCGTCGTCTTGACCAT |
| DUO1RTF | AACGTCAAACCAATCCGTCAATCC |
| DUO1RTR | CGAACAATGGCTCAGAAGAATCAGC |
| HISTONERTF | AGCTCCCTTTCCAGAGGCTA |
| HISTONERTR | TCCAAGTCTCCTACACCCAAA |
| GFPRTF | CCATCTTCTTCAAGGACGACGG |
| GFPRTR | GCAGATTGTGTGGACAGGTAATGG |
